# Free triiodothyronine in intracranial aneurysms: a sex-specific association with unruptured disease and a non-thyroidal illness signal after rupture

**DOI:** 10.3389/fneur.2026.1873351

**Published:** 2026-09-23

**Authors:** Maximilian Bschorer, Maximilian Middelkamp, Marius Mader, Shiva Sophia Nicolay, Miriam Heise, Patrick Czorlich, Lasse Dührsen, Thomas Sauvigny, Jennifer Sauvigny

**Affiliations:** 1 Department of Neurosurgery, University Medical Center Hamburg-Eppendorf, Hamburg, Germany; 2 III. Department of Medicine (Nephrology, Endocrinology), University Medical Center Hamburg-Eppendorf, Hamburg, Germany; 3 Department of Spine Surgery, Schön Klinik Hamburg Eilbek, Hamburg, Germany; 4 Division of Critical Care, Integrated Hospital Care Institute, Cleveland Clinic Abu Dhabi, Abu Dhabi, United Arab Emirates

**Keywords:** cerebrovascular risk factor, hypothyroidism, intracranial aneurysm, non-thyroidal illness syndrome, subarachnoid haemorrhage, thyroid dysfunction

## Abstract

**Introduction:**

Thyroid dysfunction has been implicated in neurovascular pathologies, but its role in intracranial aneurysm formation and rupture is uncertain. Materials and.

**Methods:**

This is a retrospective single-centre case–control study of patients with unruptured (UIA) or ruptured intracranial aneurysms (RIA) and aneurysm-free controls. Low free triiodothyronine (fT3) was defined as below 3.5 pmol/L. Multivariable logistic regression was adjusted for age, sex, hypertension, alcohol abuse, and smoking.

**Results:**

The cohort comprised 1,100 patients (60.0% female), including UIA (*n* = 213), RIA (*n* = 629), and controls (CTRL, *n* = 258). Low fT3 was observed in 58.8% of RIA patients (*p* < 0.001). In multivariable logistic regression analysis including age, sex, hypertension, alcohol abuse, and smoking, female sex (OR 4.90; 95% CI 2.70–8.92) and low fT3 (OR 3.05; 95% CI 1.28–7.28) were independently associated with UIA. Low fT3 was less frequent in UIA (3.8%) than in CTRL (14.0%) but was independently associated with UIA after risk-factor adjustment. Low fT3 was strongly associated with ruptured-aneurysm status (OR 38.0; 95% CI 15.90–90.89), a contrast likely driven by acute non-thyroidal illness syndrome after haemorrhage rather than a rupture-risk estimate. The fT3-UIA association was sex-dependent (interaction *p* = 0.030; females OR 2.43, *p* = 0.003; males OR 1.02, *p* = 0.938).

**Discussion and conclusion:**

Reduced fT3 in RIA may partly reflect impaired peripheral conversion after haemorrhage, but low fT3 was also independently associated with aneurysm presence in females. Low fT3 may be a candidate endocrine marker associated with aneurysm presence. Whether thyroid optimisation influences aneurysm development remains to be tested in prospective studies.

## Introduction

Intracranial aneurysms affect an estimated 2–5% of the general population (1). Rising detection rates are likely driven, in part, by the wider availability of angiographic imaging (2). Although most patients remain asymptomatic, aneurysmal rupture leading to subarachnoid haemorrhage (aSAH) is catastrophic. Despite recent advances in neurocritical care and surgical or endovascular therapies, the prognosis following aSAH remains poor; case fatality rates approach 30–40%, and survivors often endure persistent neurological deficits, cognitive impairment, and reduced quality of life (3).

There has been a shift from uniform to risk-stratified management of unruptured intracranial aneurysms, driven by the recognition that most such lesions will never rupture while preventive treatment carries non-trivial procedural morbidity (4). The PHASES score, derived from data on 8,382 patients with 230 rupture events, estimates 5-year rupture risk from 0.4 to 17.8% on the basis of six clinical and radiographic variables and remains the most widely used clinical instrument (5). The complementary Unruptured Intracranial Aneurysm Treatment Score (UIATS) integrates aneurysm- and patient-related factors to explicitly weigh preventive treatment against conservative follow-up, which is an aspect that PHASES does not address (6). Long-term external validation in a Finnish cohort followed without treatment-selection bias has since demonstrated that cigarette smoking, absent from the original PHASES derivation, carries independent predictive weight, motivating refined treatment scores that incorporate smoking, age, aneurysm size, and location (7). More recently, machine-learning algorithms trained on routine health-examination variables have extended this assessment process, identifying high-risk individuals within the general population (8). Despite this progress, existing scores remain anchored almost exclusively in demographic, anatomic, and conventional vascular-risk variables. Candidate endocrine factors are not represented in these current algorithms.

Thyroid disease, especially hypothyroidism, is among the most prevalent endocrine disorders globally, with many cases remaining undiagnosed or untreated for prolonged periods (9, 10). In Europe, approximately 5% of the population has undiagnosed hypothyroidism (9). Hypothyroidism alters lipid metabolism, vascular resistance, endothelial function, and arterial stiffness, which are changes that may weaken the cerebrovascular wall (11, 12). In the context of critical illness, such as aSAH, patients frequently exhibit non-thyroidal illness syndrome (NTIS), a condition characterised by low serum triiodothyronine (fT3) levels in the absence of a primary thyroid disorder, typically arising as an adaptive endocrine response to severe physiological stress (13, 14).

Recent epidemiological data support an association between hypothyroidism and intracranial aneurysms (15). A population-based case–control study by Park et al. identified a sex-specific association, demonstrating that hypothyroidism in females was associated with an increased likelihood of unruptured cerebral aneurysms (16). The few studies addressing the link between thyroid dysfunction and intracranial aneurysm pathology have produced conflicting results (17). Measured thyroid hormone levels, as opposed to coded diagnoses, have rarely been examined in this context. The aim of this study was therefore to assess whether measured thyroid hormone status, and particularly fT3, is associated with the presence of unruptured intracranial aneurysms and with ruptured-aneurysm status in a single-centre cohort.

## Materials and methods

### Study design and population

This retrospective, single-centre case–control study was conducted at the Department of Neurosurgery, University Medical Center Hamburg-Eppendorf, Germany, with patients with ruptured or unruptured intracranial aneurysms and aneurysm-free controls identified from the same institutional population. We screened patients treated between 2010 and 2023 for whom thyroid function parameters (TSH, fT3, and fT4) and medical history regarding thyroid disease were available. The study population included patients with radiological evidence of ruptured or unruptured intracranial aneurysms, as well as a control group without aneurysms. This study is reported according to the STROBE guidelines for case–control studies.

Patients lacking radiological neurovascular imaging were excluded. All inclusion, exclusion, and blood-sampling procedures applied identically to male and female patients; no sex-specific sampling procedure was used, and sex-stratified comparisons were performed only at the analysis stage. The inclusion criteria for the ruptured intracranial aneurysm (RIA) group were as follows: (1) laboratory assessment of thyroid hormone levels within 24 h of hospital admission and (2) radiological confirmation of a ruptured aneurysm requiring surgical or endovascular treatment. Treatment of the aneurysm was a condition of inclusion in the RIA group, excluding patients too severely affected or unstable for treatment. Patients with unruptured intracranial aneurysms (UIA) were included if thyroid function test results were available in the institutional laboratory database. Individuals with a history of aSAH were excluded from the UIA cohort. The control group (CTRL) comprised patients treated at the neurological or neurosurgical department who underwent magnetic resonance angiography or digital subtraction angiography with no evidence of intracranial aneurysms. Only individuals with available thyroid function tests and no history of aSAH were included. Controls were selected from the same institution to minimise interlaboratory assay variability.

### Outcome measures and independent variables

Serum levels of thyroid-stimulating hormone (TSH), fT3, and free thyroxine (fT4) were analysed in a certified institutional laboratory. The local reference ranges were as follows: TSH, 0.55–7.78 mU/L; fT3, 3.5–6.5 pmol/L; and fT4, 11.5–22.7 pmol/L (Figure 1A). A reduced fT3 level (<3.5 pmol/L) was defined as low fT3. Demographic and clinical variables, including age, sex, hypertension, alcohol abuse, and smoking status, were collected from electronic medical records. Levothyroxine-treated patients with low fT3 are patients on thyroid replacement therapy who exhibited a low fT3 level, a descriptive label that does not assert a specific cause, since low fT3 may also arise from non-thyroidal illness, inflammation, or nutritional status. Untreated subclinical hypothyroidism is characterised by elevated TSH with normal fT4 levels in the absence of medication, whereas subclinical hyperthyroidism presents as suppressed TSH with normal circulating thyroid hormones. Finally, overt hyperthyroidism is defined by suppressed TSH accompanied by elevated levels of fT4 and/or fT3.

**Figure 1:**
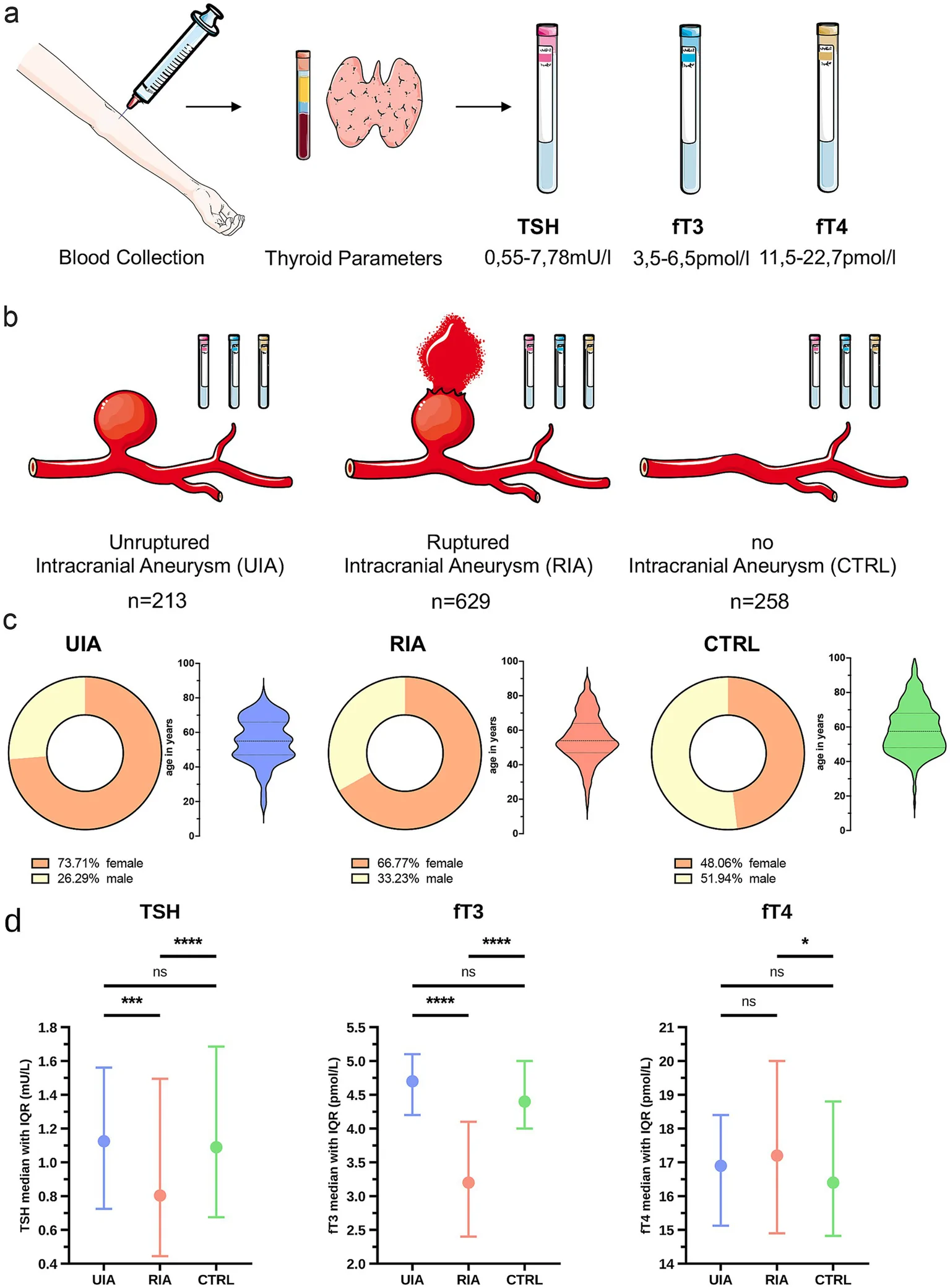
Study workflow and cohort characteristics. **(A)** Overview of the workflow for blood collection and determination of thyroid hormone parameters (TSH, fT3, and fT4) in serum samples. **(B)** Distribution of study participants among the cohorts: unruptured intracranial aneurysm (UIA, *n* = 213), ruptured intracranial aneurysm (RIA, *n* = 629), and controls without intracranial aneurysms (CTRL, *n* = 258). **(C)** Demographic characteristics showing age and sex distribution within each group (UIA, RIA, CTRL). **(D)** Comparative analysis of serum TSH, fT3, and fT4 levels across the three groups (UIA, RIA, CTRL). Between-group comparisons in panel **(D)** are pairwise Mann–Whitney U tests. Statistical significance is indicated by asterisks, where *p < 0.05, **p < 0.01, ***p < 0.001, and ****p < 0.0001; ns, not significant (*p* > 0.05). CTRL, controls; fT3, free triiodothyronine; fT4, free thyroxine; ns, not significant; RIA, ruptured intracranial aneurysm; TSH, thyroid-stimulating hormone; UIA, unruptured intracranial aneurysm.

### Statistical analysis

Distributional shape was assessed for every continuous variable before analysis. Variables with an approximately symmetric distribution are summarised as mean ± standard deviation and were compared across groups by one-way analysis of variance. Variables with a skewed distribution are summarised as median with interquartile range and were compared across the three groups by the Kruskal-Wallis test, with pairwise Mann–Whitney U tests for individual between-group contrasts. Categorical variables are reported as the number of patients with the characteristic over the number with available data, together with the percentage, and were compared by the chi-square test, or by the Fisher’s exact test where any expected cell count was below five. Multivariable logistic regression models were used to calculate odds ratios (ORs) with 95% confidence intervals (CIs), adjusted for age, sex, and relevant comorbidities. The threshold for statistical significance was a 2-sided *p* value < 0.05. Pairwise deletion was applied to handle missing data. Statistical analyses were performed using GraphPad Prism (version 10.0; GraphPad Software, San Diego, CA) and IBM SPSS Statistics 30.0 (IBM Corp, Armonk, NY). Sensitivity analyses (alternative fT3 cutoffs, levothyroxine exclusion, missing-data analysis, propensity-score matching, hypertension and age interactions) are reported in the Supplementary material. No adjustment for multiple comparisons was applied, and all subgroup, interaction, quartile, and nested-model analyses are exploratory. The covariates age, sex, hypertension, smoking, and alcohol abuse were pre-specified on clinical grounds and prior literature rather than by data-driven selection. Age was modelled as a continuous variable; fT3 was analysed primarily as a dichotomous variable at the laboratory threshold of 3.5 pmol/L and, in supporting analyses, continuously and by quartile. Linearity of the logit for continuous predictors was assessed with the Box-Tidwell test and was satisfied for age and fT3, and influential observations were evaluated using Cook distance. As this was a retrospective analysis of all eligible patients, no *a priori* sample-size calculation was performed; a *post hoc* assessment indicated limited power for the low-fT3 UIA comparison given the small number of exposed cases, and these analyses are therefore considered hypothesis-generating.

### Ethics approval

This study was approved by the Ethics Committee of the Hamburg Medical Association (Ärztekammer Hamburg, Germany; reference 2025-101644-BO-ff) and was conducted in accordance with the Declaration of Helsinki. Written informed consent was waived due to the retrospective analysis of anonymised patient data.

## Results

### Study population

A total of 1,100 patients were included in the analysis, comprising 213 patients with unruptured intracranial aneurysms (UIA), 629 patients with ruptured intracranial aneurysms (RIA), and 258 control subjects without evidence of intracranial aneurysms (CTRL) (Figures 1B,C). Baseline characteristics are detailed in Table 1. Mean age was 55.4 ± 13.1 years in the UIA group, 55.4 ± 13.7 years in the RIA group, and 58.7 ± 14.0 years in the CTRL group, and differed significantly across the three groups (*p* = 0.004). Females accounted for 73.7% of the UIA group, 66.8% of the RIA group, and 48.1% of the CTRL group, and the sex distribution likewise differed significantly across groups (*p* < 0.001). A history of smoking was significantly more prevalent in the RIA group (23.3%) compared to the UIA (10.3%) and CTRL (14.9%) groups (*p* < 0.001). Similarly, alcohol abuse was more frequent in the RIA group (5.9%) compared to the other cohorts (*p* = 0.013). No significant differences were observed in the prevalence of hypertension (*p* = 0.309) or the use of thyroid replacement therapy (*p* = 0.148). The control group consisted of patients evaluated for the following indications: idiopathic Parkinson disease (*n* = 111, 42.9%), suspected stroke (*n* = 58, 22.4%), epilepsy (*n* = 24, 9.3%), cervical dystonia (*n* = 11, 4.3%), and patients with no underlying pathological diagnosis on imaging (*n* = 55, 21.2%).

**Table 1:** Clinical and demographic characteristics.

Characteristic	Ruptured intracranial aneurysm (*n* = 629)	Unruptured intracranial aneurysm (*n* = 213)	No aneurysm (*n* = 258)	*p* value
Age (yr) — mean ± SD	55.43 ± 13.65	55.39 ± 13.11	58.66 ± 13.97	**0.004**
Male Sex — no. (%)	209 (33.2)	56 (26.3)	134 (51.9)	**< 0.001**
Medical history — no. (%)				
Hypertension	266 (43.1)	102 (47.9)	34 (39.1)	0.309
Thyroid replacement therapy	67 (11.5)	34 (16.0)	27 (10.5)	0.148
Smoking history	143 (23.3)	22 (10.3)	13 (14.9)	**< 0.001**
Alcohol abuse	36 (5.9)	2 (0.9)	4 (4.6)	**0.013**
Laboratory values — median [IQR]				
TSH (mU/L)	0.80 [0.45–1.50]	1.13 [0.73–1.56]	1.09 [0.68–1.69]	**< 0.001**
fT4 (pmol/L)	17.20 [14.90–20.00]	16.90 [15.13–18.40]	16.40 [14.83–18.80]	**0.047**
fT3 (pmol/L)	3.20 [2.40–4.10]	4.70 [4.20–5.10]	4.40 [4.00–5.00]	**< 0.001**
Thyroid function status — no./total (%)				
Euthyroid	72/255 (28.2)	123/157 (78.3)	77/107 (72.0)	**< 0.001**
Subclinical hypothyroidism	3/255 (1.2)	0/157 (0.0)	0/107 (0.0)	**0.311**
Subclinical hyperthyroidism	96/255 (37.6)	30/157 (19.1)	20/107 (18.7)	**< 0.001**
Overt hyperthyroidism	1/255 (0.4)	0/157 (0.0)	0/107 (0.0)	1.000
Low fT3 (<3.5 pmol/L)	150/255 (58.8)	6/157 (3.8)	15/107 (14.0)	**< 0.001**
Low fT3, levothyroxine-treated	19/255 (7.5)	1/157 (0.6)	4/107 (3.7)	**0.003**

Age, sex distribution, thyroid hormone parameters (TSH, fT3, fT4), prevalence of low fT3 (<3.5 pmol/L), use of thyroid drugs, smoking status, alcohol abuse, and hypertension in patients with unruptured intracranial aneurysm (UIA, *n* = 213), ruptured intracranial aneurysm (RIA, *n* = 629), and controls (CTRL, *n* = 258). Percentages are calculated from patients with available data. All rows in the Thyroid Function Status block use a single denominator per group, namely the patients with both a TSH and an fT3 result available (RIA *n* = 255, UIA *n* = 157, CTRL *n* = 107). The categories in that block are not mutually exclusive, because low fT3 and subclinical hyperthyroidism occur in the same patient in 64 RIA, 2 UIA, and 5 CTRL cases, which is the expected biochemical pattern of non-thyroidal illness syndrome. Euthyroid denotes patients with none of the listed abnormalities. Values in bold are statistically significant (*p* < 0.05).

### Thyroid function parameters

Median serum TSH was 1.13 mU/L (IQR 0.73–1.56) in the UIA group, 0.80 mU/L (IQR 0.45–1.50) in the RIA group, and 1.09 mU/L (IQR 0.68–1.69) in the CTRL group and differed significantly across the three groups (*p* < 0.001). This difference is attributed entirely by the ruptured group, in which TSH was lower than in both the UIA group (*p* < 0.001) and the CTRL group (*p* < 0.001), whereas the UIA and CTRL groups did not differ from each other (*p* = 0.999). Median fT3 was likewise lowest in the RIA group at 3.20 pmol/L (IQR 2.40–4.10), compared with 4.70 pmol/L (IQR 4.20–5.10) in the UIA group and 4.40 pmol/L (IQR 4.00–5.00) in the CTRL group (*p* < 0.001 for both pairwise comparisons with RIA). Median fT3 did not differ significantly between the CTRL and UIA groups (*p* = 0.051). Median fT4 was 17.20 pmol/L (IQR 14.90–20.00) in the RIA group, 16.90 pmol/L (IQR 15.13–18.40) in the UIA group, and 16.40 pmol/L (IQR 14.83–18.80) in the CTRL group, a modest overall difference (*p* = 0.047) that reached nominal significance only for the RIA versus CTRL contrast (*p* = 0.041) (Figure 1D). The prevalence of low fT3 (defined as fT3 < 3.5 pmol/L) was significantly higher in the RIA group (58.8%) than in the UIA (3.8%) and CTRL groups (14.0%) (*p* < 0.001).

### Multivariable analysis

In multivariable logistic regression comparing UIA with controls (Figure 2), female sex showed the strongest independent association with the presence of UIA (OR 4.90; 95% CI 2.70–8.92; *p* < 0.001). Adjusted for age, sex, and neurovascular risk factors, low fT3 was associated with a significant increase in the likelihood of UIA presence (OR 3.05; 95% CI 1.28–7.28; *p* = 0.012). Age, hypertension, smoking history, and alcohol abuse did not reach statistical significance in this comparison (*p* > 0.05).

**Figure 2:**
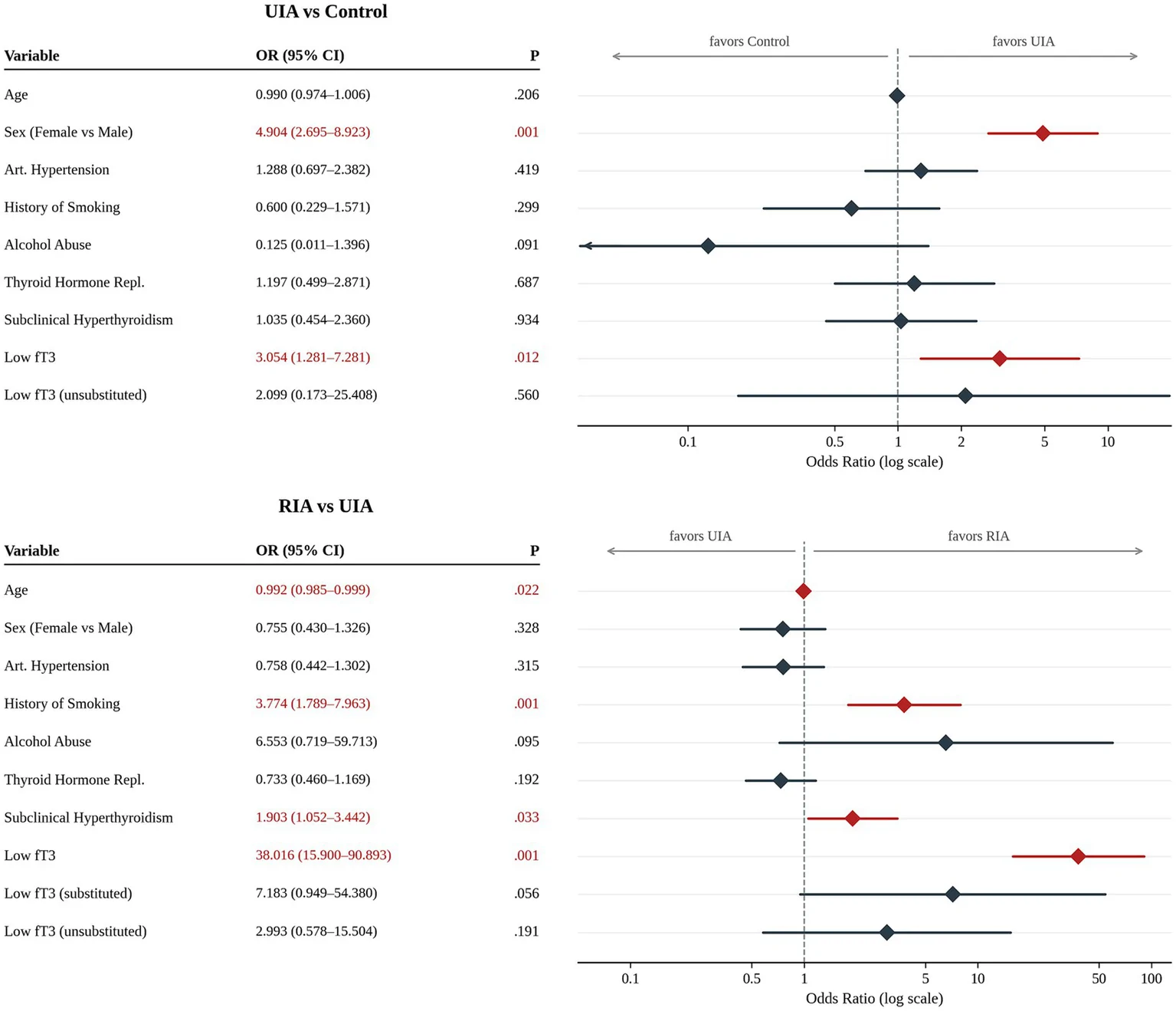
Multivariable logistic regression analysis. Multivariable logistic regression analysis comparing UIA vs. CTRL group (top) and RIA vs. UIA (bottom). The odds ratio for low fT3 is derived from a model adjusted for age, sex, hypertension, alcohol abuse, and history of smoking. The remaining thyroid categories are displayed alongside for comparison. Because they overlap with low fT3 and with one another, their odds ratios should not be read as mutually independent thyroid contributions. Male sex is the reference category for the sex term, so the odds ratio shown for sex is that of female relative to male sex. Low fT3 denotes a measured free triiodothyronine level below 3.5 pmol/L. CTRL, controls; fT3, free triiodothyronine; RIA, ruptured intracranial aneurysm; UIA, unruptured intracranial aneurysm.

Low fT3 was significantly associated with ruptured-aneurysm status in the RIA vs. UIA comparison (OR 38.0; 95% CI 15.90–90.89; *p* < 0.001) (Figure 2). Subclinical hyperthyroidism was also associated with rupture in this model (OR 1.90; 95% CI 1.05–3.44; *p* = 0.033), although this category overlapped substantially with low fT3 (64 RIA patients met both definitions), so it should not be read as an independent thyroid contribution. Among traditional risk factors, a history of smoking was independently associated with rupture (OR 3.77; 95% CI 1.79–7.96; *p* < 0.001). In contrast to the UIA vs. Control analysis, sex was not significantly associated with rupture in this model (OR 0.76; *p* = 0.328). Neither age nor hypertension showed a significant independent association with rupture in this multivariable adjustment.

### Subgroup analysis

Given the strong female preponderance observed in UIA, a subgroup analysis was performed. A significant fT3-sex interaction was identified (*p* = 0.030), indicating that the effect of low fT3 on aneurysm presence differed between sexes. In sex-stratified models, low fT3 was significantly associated with UIA in females (OR 2.43; 95% CI 1.34–4.40; *p* = 0.003) but not in males (OR 1.02; 95% CI 0.62–1.67; *p* = 0.938) (Figure 3B; Table 2). An exploratory quartile-based analysis of continuous fT3 levels in the UIA vs. CTRL comparison showed higher odds of UIA in some lower-fT3 categories, although the pattern was not monotonic and only the lowest and third quartiles reached significance (Q3 vs. Q4: OR 2.39, *p* = 0.033; Q1 vs. Q4: OR 2.54, *p* = 0.031; P for trend = 0.015). (Figure 3C). Receiver operating characteristic analysis showed that adding fT3 to a base model of age, sex, and hypertension improved the area under the curve from 0.693 to 0.731 (ΔAUC = 0.038), although the likelihood ratio test did not reach statistical significance (*p* = 0.064). Adding TSH and fT4 on top of fT3 produced no further improvement and marginally reduced discrimination (AUC 0.728) (Figure 3D).

**Figure 3:**
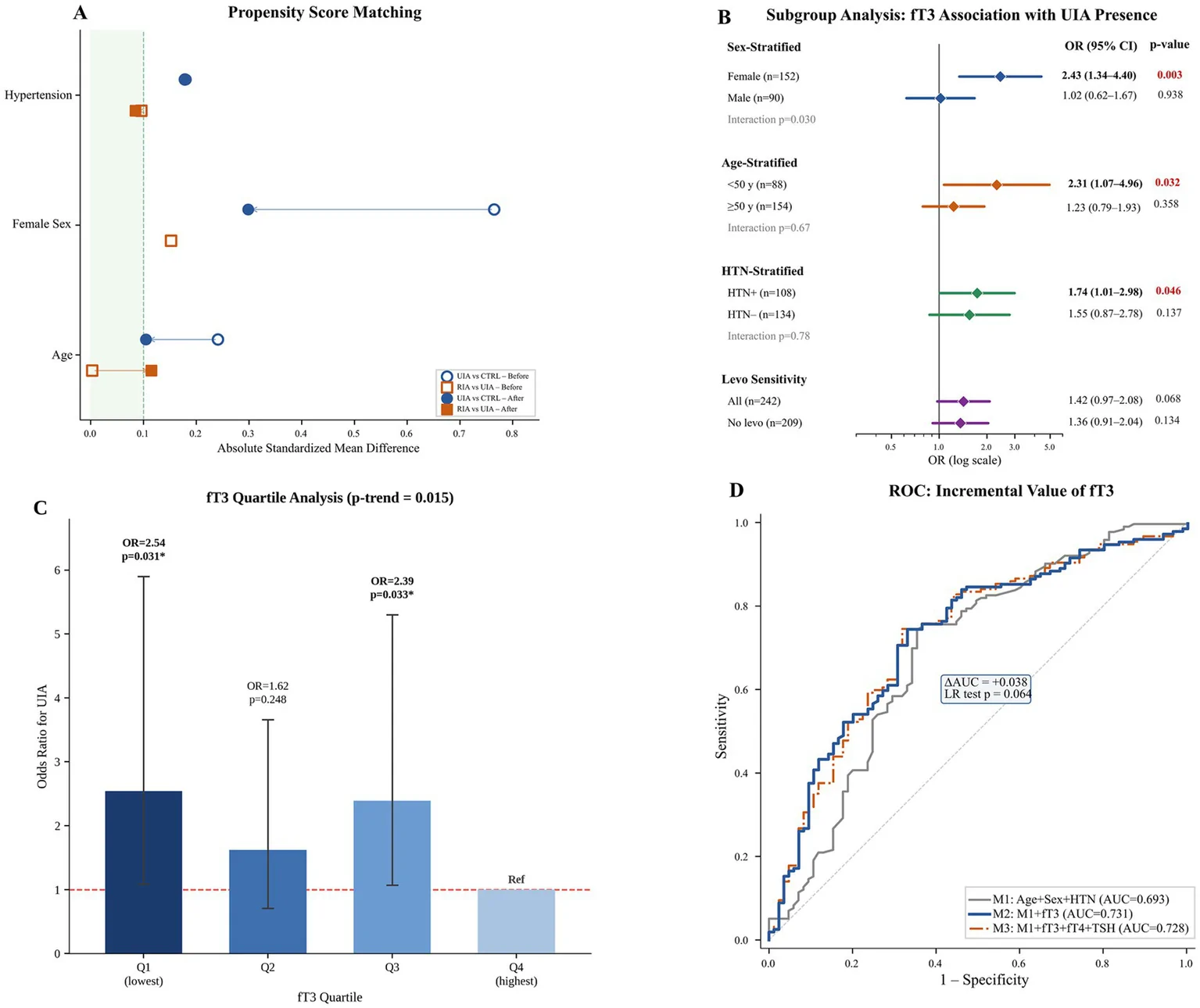
fT3-UIA association. **(A)** Love plot showing standardised mean differences (SMD) for covariates before and after 1:1 propensity-score matching, shown for both the UIA versus CTRL and the RIA versus UIA comparisons. The dashed vertical line at SMD = 0.1 indicates the conventional threshold for acceptable balance. **(B)** Forest plot of subgroup analyses showing odds ratios for fT3 < 3.5 pmol/L across sex-stratified, age-stratified (<50 vs. ≥ 50 years), hypertension-stratified, and levothyroxine-sensitivity models. **(C)** Exploratory quartile analysis of continuous fT3 levels (UIA vs. CTRL), with odds ratios referenced to the highest quartile (Q4, 5.20–6.70 pmol/L). Quartiles are displayed in sequential order from Q1 (lowest fT3) to Q4 (highest fT3). Error bars represent 95% confidence intervals; the dashed line indicates the null (OR = 1). **(D)** Receiver operating characteristic (ROC) curves comparing 3 nested logistic regression models for UIA vs. CTRL discrimination: Model 1 (age, sex, hypertension), Model 2 (Model 1 + fT3), and Model 3 (Model 2 + TSH + fT4). AUC, area under the curve; CTRL, controls; fT3, free triiodothyronine; fT4, free thyroxine; ROC, receiver operating characteristic; SMD, standardised mean difference; TSH, thyroid-stimulating hormone; UIA, unruptured intracranial aneurysm.

**Table 2:** Subgroup and exploratory quartile analyses for the association of low fT3 with unruptured intracranial aneurysm presence (UIA vs. CTRL).

Analysis	Subgroup	*N*	OR (95% CI)	*p* value
Sex-stratified subgroup analysis (UIA vs. CTRL)				
fT3 × Sex interaction	—	**242**	**2.32 (1.08–4.96)**	**0.030**
Low fT3 (<3.5 pmol/L)	Females	**152**	**2.43 (1.34–4.40)**	**0.003**
Age (per year)	Females		1.00 (0.97–1.03)	0.833
Hypertension	Females		0.60 (0.26–1.37)	0.225
Low fT3 (<3.5 pmol/L)	Males	90	1.02 (0.62–1.67)	0.938
Age (per year)	Males		0.98 (0.94–1.02)	0.250
Hypertension	Males		2.34 (0.92–5.95)	0.073
fT3 quartile dose–response analysis (UIA vs. CTRL)				
Q4 (reference)	5.20–6.70 pmol/L	54	1.00 (ref)	—
Q3	**4.70–5.10 pmol/L**	**64**	**2.39 (1.07–5.30)**	**0.033**
Q2	4.30–4.60 pmol/L	50	1.62 (0.71–3.66)	0.248
Q1 (lowest)	**0.90–4.20 pmol/L**	**74**	**2.54 (1.09–5.90)**	**0.031**
P for trend	**—**		**—**	**0.015**

The sex-stratified analysis was derived from multivariable logistic regression models that included an fT3 x sex interaction term; stratified models were adjusted for age, hypertension, smoking, and alcohol abuse. The quartile analysis used continuous fT3 levels categorised into quartiles, with Q4 (highest fT3) as the reference group; models were adjusted for age, sex, hypertension, smoking, and alcohol abuse. P for trend was derived from an ordinal quartile variable. The quartile pattern was not monotonic and is presented as exploratory. CTRL, controls; fT3, free triiodothyronine; UIA, unruptured intracranial aneurysm. Values in bold are statistically significant (p < 0.05).

When thyroid function was classified categorically (euthyroid, subclinical hypothyroidism, subclinical hyperthyroidism, and overt dysfunction), no significant difference in thyroid state distribution was observed between the UIA and CTRL groups (*p* = 1.000), with approximately 92% euthyroidism in both groups. In contrast, the RIA group showed a significantly different thyroid state profile compared to UIA (*p* < 0.001). Full details of these analyses, including the propensity-score matched cohort, the continuous fT3 model, the nested ROC model comparison, the hypertension x fT3 interaction, and the age-stratified analysis, are provided in the Supplementary material.

## Discussion

In this case–control cohort of 1,100 patients, low serum fT3 was independently associated with the presence of unruptured intracranial aneurysms (UIA vs. CTRL; OR 3.05) and was very strongly associated with rupture (RIA vs. UIA; OR 38.0). The UIA association was confined to females (interaction *p* = 0.030), pointing to a sex-specific endocrine contribution to aneurysm susceptibility. Although univariate analysis showed a higher prevalence of low fT3 in controls (14.0%) than in the UIA group (3.8%), this pattern reversed after multivariate adjustment for age, sex, and neurovascular risk factors, with low fT3 independently associated with UIA presence (*p* = 0.012). In an analysis of ICD-coded diagnoses, Park et al. reported a directionally consistent finding, with higher UIA incidence in hypothyroid patients (16). Mechanistically, thyroid hormones are central to vascular integrity (18). Free T3 modulates vascular endothelial function and extracellular matrix turnover, and low fT3 has been linked to impaired arterial wall repair, increased vascular stiffness, and a proinflammatory milieu, which may promote aneurysm formation (15, 19). Consistent with this, in patients with acute ischemic stroke, lower fT3 has been shown to correlate with higher inflammatory markers and greater clinical severity (20).

The fT3-UIA association was sex-dependent (interaction *p* = 0.030). The association between low fT3 and UIA presence was observed only in females (OR 2.43; males OR 1.02), consistent with known sex differences in thyroid physiology (10). This sex interaction rests on a small number of exposed cases and should be regarded as hypothesis-generating. Declining oestrogen and fT3 may act together to weaken the vascular wall, as both modulate endothelial nitric oxide synthase expression (21–23), though this mechanism is hypothesis-generating and unmeasured factors including menopausal status, oestrogen therapy, and body mass index could not be accounted for. The exploratory quartile analysis showed higher UIA odds in some lower-fT3 categories but was not monotonic, so it should not be read as a dose–response relationship. Adding fT3 to conventional risk factors improved discrimination (ΔAUC = 0.038) but did not reach statistical significance (likelihood ratio test *p* = 0.064). Adding TSH and fT4 on top of fT3 gave no further gain and marginally reduced discrimination (AUC 0.728 versus 0.731). Within the thyroid panel the discriminative signal therefore appears to be carried by fT3 specifically rather than by thyroid function testing in general, which is consistent with fT3 rather than the panel as a whole being the candidate marker.

The strong association between low fT3 and aneurysm rupture (RIA vs. UIA, OR 38.0) raises the question of whether low fT3 drives rupture or is a consequence of it. Casulari et al. observed significantly lower fT3 and fT4 levels in RIA patients within 24 h of rupture, consistent with NTIS (24). This suppression of peripheral T4-to-T3 conversion is likely driven by the surge in cortisol and cytokines associated with the haemorrhage itself (13, 25). In the present cohort, median TSH in the ruptured group was also lower than in the unruptured and control groups (0.80 versus 1.13 and 1.09 mU/L; *p* < 0.001), so the ruptured group showed low fT3 together with non-elevated TSH, which is the pattern that characterises the syndrome rather than primary hypothyroidism. Given that blood samples were obtained within 24 h of haemorrhage, the observed OR almost certainly reflects confounding by NTIS, which cannot be disentangled from pre-existing thyroid dysfunction in the present study design.

Subclinical hyperthyroidism was also associated with rupture in the multivariable model (RIA vs. UIA, OR 1.90). This finding should be interpreted with caution and is unlikely to represent an independent thyroid contribution. The categories of subclinical hyperthyroidism and low fT3 were not mutually exclusive, and 64 ruptured-aneurysm patients met both definitions, reflecting the characteristic biochemical pattern of acute NTIS in which suppressed TSH accompanies low peripheral fT3 (13, 24). The OR 1.90 therefore largely re-expresses the same post-haemorrhage NTIS signal captured by the low-fT3 finding, rather than an additional risk factor. On the unruptured side, Park and colleagues observed lower UIA prevalence in patients with hyperthyroidism (adjusted OR 0.71; 95% CI 0.54–0.93), but their register-based analysis used ICD-coded diagnoses and could not distinguish overt from subclinical disease (16).

These findings raise several hypotheses for future work rather than immediate clinical recommendations. First, whether fT3 adds value to existing risk-stratification instruments such as the PHASES score or the Unruptured Intracranial Aneurysm Treatment Score (UIATS) is a question for prospective study; our incremental-discrimination analysis did not reach significance, so the present data do not support incorporating fT3 into these tools or recommending routine fT3 measurement in clinical practice (5, 6, 8). Second, Said and colleagues found that thyroid hormone replacement therapy may improve outcomes after aneurysm rupture (17). Further trials are needed to test whether preemptive correction of hypothyroidism mitigates aneurysm growth or rupture.

## Limitations

The large cohort (*n* = 1,100) provided adequate power to examine associations across thyroid states, though several limitations warrant discussion. Limitations include the retrospective nature of the analysis and the composition of the control group. As controls were recruited from neurological or neurosurgical services, the overrepresentation of neurological comorbidities may limit the generalisability of these findings to the broader population. It was not possible to definitively separate pre-existing chronic hypothyroidism from acute stress-induced low fT3 (NTIS) in the ruptured group, as pre-rupture thyroid levels were not available. The disruption of the hypothalamic–pituitary-thyroid axis by the haemorrhage itself (eg, via increased intracranial pressure or cortisol-mediated suppression) confounds the causal interpretation of post-admission laboratory values (26).

The institutional TSH reference range (0.55–7.78 mU/L) is wider than contemporary guideline-based ranges (typically 0.4–4.5 mU/L), which may affect classification of thyroid states and limit comparability with existing literature (27). In a sensitivity analysis reclassifying thyroid status with a guideline-based TSH upper limit of 4.5 mU/L, only a small number of patients shifted category in each group and the fT3-based associations were unchanged, because the fT3 cut-off is independent of the TSH range. A further limitation is indication bias, since cohort entry required an available thyroid panel and clinicians order such panels for specific reasons; pairwise deletion of missing values may compound this, and the direction of the resulting bias cannot be fully predicted. The control group is also a disease-enriched neurology sample rather than a neutral comparator, and fT3 by control diagnosis (Supplementary material) shows that the low-fT3 burden is concentrated in the suspected-stroke and no-defined-pathology subgroups. Propensity-score matching, in which all post-matching standardised mean differences were below 0.1 (Figure 3A), balances measured covariates only. While the analysis adjusted for major cardiovascular risk factors, residual confounding from unmeasured variables, including body mass index, oestrogen status, and specific neurological comorbidities in the control group, cannot be excluded.

One caveat applies collectively to all of the exploratory analyses reported here. The sex-, age- and hypertension-stratified subgroup models, the fT3 by sex and fT3 by hypertension interaction terms, the quartile analysis of continuous fT3, and the nested ROC model comparisons were all performed in addition to the pre-specified primary comparisons, and none was adjusted for multiple testing. All should therefore be read as hypothesis-generating rather than confirmatory, and their nominal *p* values do not carry the usual interpretation.

## Conclusion

Low fT3 was independently associated with unruptured intracranial aneurysms in females, suggesting a candidate endocrine marker of aneurysm susceptibility that warrants further investigation. The OR 38.0 signal for rupture cannot be separated from acute non-thyroidal illness syndrome in this design and should not be interpreted as causal. Prospective cohorts with pre-rupture thyroid measurements are needed to determine whether fT3 adds incremental value to existing risk-stratification tools and whether thyroid optimisation alters aneurysm natural history.

## Data Availability

The datasets presented in this article are not readily available because the data can be made available from the corresponding author upon reasonable request and subject to a formal data-sharing agreement with the University Medical Center Hamburg-Eppendorf. Requests to access the datasets should be directed to maximilian.tennis@gmail.com.
