# Supplementary material for: Free triiodothyronine in intracranial aneurysms: a sex-specific association with unruptured disease and a non-thyroidal illness signal after rupture
Source: Front Neurol. 2026 Sep 23;17:1873351. doi: 10.3389/fneur.2026.1873351 (PMC13642133; doi:10.3389/fneur.2026.1873351)
Supplement: Supplementary file 1 [file Supplementary_file_1.DOCX]

**Supplementary Material**

# Supplementary Methods

All multivariable logistic regression models were adjusted for age, sex, hypertension, smoking, and alcohol abuse unless stated otherwise. Sensitivity, specificity, and odds ratios for alternative fT3 cutoffs were calculated against the primary outcome (UIA presence). Propensity-score matching (PSM) was performed using 1:1 nearest-neighbour matching with a logit-distance calliper of 0.2 standard deviations. Balance was assessed using standardised mean differences (SMD), with SMD < 0.1 considered acceptable. Receiver operating characteristic (ROC) analysis compared three nested logistic regression models for UIA versus CTRL discrimination, with incremental likelihood-ratio tests. All P values are two-sided.

# 1. Sensitivity Analyses

**Supplementary Table S1.** Robustness of fT3 cutoff for UIA vs CTRL

*Multivariable logistic regression adjusted for age, sex, hypertension, smoking, and alcohol abuse. The association between low fT3 and UIA presence remained significant across all tested cutoffs (3.0-4.5 pmol/L), with the strongest effect at the most stringent threshold (<3.0 pmol/L, OR 3.72).*

| fT3 cutoff (pmol/L) | N(low) | OR (95% CI) | P value | Sensitivity | Specificity |
| --- | --- | --- | --- | --- | --- |
| <3.0 | 28 | 3.72 (1.23-11.24) | 0.020 | 0.08 | 0.98 |
| <3.5 (primary) | 68 | 3.05 (1.28-7.28) | 0.012 | 0.19 | 0.93 |
| <4.0 | 127 | 2.21 (1.15-4.25) | 0.017 | 0.34 | 0.83 |
| <4.5 | 187 | 1.89 (1.07-3.35) | 0.029 | 0.49 | 0.73 |

**Supplementary Table S2.** Sensitivity analysis excluding levothyroxine users

*Multivariable logistic regression adjusted for age, sex, hypertension, smoking, and alcohol abuse. Excluding patients on levothyroxine replacement therapy did not materially change the association (OR 3.05 to 2.87).*

| Model | N | fT3 OR (95% CI) | P value | Excluded |
| --- | --- | --- | --- | --- |
| Full cohort | 242 | 3.05 (1.28-7.28) | 0.012 | - |
| Excl. levothyroxine | 218 | 2.87 (1.14-7.21) | 0.025 | 24 (9.9%) |

**Supplementary Table S3.** Missing data analysis for fT3 measurements

*NS, not significant. No significant differences in age, sex, or hypertension were observed between patients with and without an available fT3 measurement. Availability of fT3 did, however, differ markedly between study groups (Supplementary Table S13), so missingness should not be assumed to be completely at random, and bias arising from the clinical indication for thyroid testing cannot be excluded.*

| Variable | fT3 available | fT3 missing | P value | Test | Interpretation |
| --- | --- | --- | --- | --- | --- |
| Age (years) | 52.3 ± 14.2 | 54.1 ± 15.8 | 0.112 | Mann-Whitney | NS |
| Sex (% female) | 63.0% | 58.4% | 0.201 | Chi-square | NS |
| HTN (% yes) | 41.2% | 43.7% | 0.478 | Chi-square | NS |

**Supplementary Figure S1.** Forest plot of sensitivity analyses for the association between low fT3 and ruptured-aneurysm status (RIA vs UIA), covering the primary analysis, propensity-score matching, levothyroxine exclusion, era stratification, and alternative fT3 cutoff thresholds. Multivariable logistic regression odds ratios for low fT3 (<3.5 pmol/L) under sequential model variations: full cohort, excluding levothyroxine users, propensity-score matched, complete-case, and alternative cutoff thresholds. Effect estimates are directionally consistent across all sensitivity analyses.

***Alt text:*** *Forest plot listing five sensitivity analyses on the y-axis with point estimates and 95% confidence intervals on a log-scaled x-axis; all confidence intervals exclude one and the estimates cluster between 2.5 and 3.7.*

**Supplementary Figure S2.** Violin plot of fT3 distribution by treatment era (UIA vs CTRL).

**Supplementary Figure S3.** Distribution of thyroid hormone values by study group. Violin plot with overlaid box plot and individual observations for TSH, fT3, and fT4 in patients with unruptured intracranial aneurysm (UIA), ruptured intracranial aneurysm (RIA), and controls (CTRL). Shading marks the institutional laboratory reference range for each hormone (TSH 0.55-7.78 mU/L, fT3 3.5-6.5 pmol/L, fT4 11.5-22.7 pmol/L), and the dashed line in the fT3 panel marks the 3.5 pmol/L low-fT3 threshold. Boxes show the median and interquartile range, whiskers extend to 1.5 times the interquartile range, and each point is one patient. Group sizes reflect the number of patients with the relevant measurement available. Between-group comparisons are Kruskal-Wallis tests.

Distribution of free triiodothyronine concentrations across two-year intervals from 2010 to 2023 in UIA and CTRL groups. No systematic temporal trend was observed in either cohort, indicating that the fT3-UIA association is unlikely to be confounded by changes in laboratory assay calibration over the study period.

***Alt text:*** *Series of violin plots showing fT3 distributions over time intervals for UIA and CTRL groups, with overlapping medians and interquartile ranges across eras.*

# 2. Propensity-Score Matching

**Supplementary Table S4.** Propensity-score matched analysis (UIA vs CTRL)

*SMD, standardised mean difference. 1:1 nearest-neighbour matching with logit-distance calliper (0.2 x SD); balance threshold SMD < 0.1. After matching, all covariates achieved acceptable balance, and the association between low fT3 and UIA presence remained significant (OR 2.73, P = 0.034).*

| Variable | Before PSM | After PSM | SMD after | Balance |
| --- | --- | --- | --- | --- |
| Age | SMD = 0.34 | SMD = 0.05 | 0.05 | Balanced |
| Sex (female) | SMD = 0.62 | SMD = 0.03 | 0.03 | Balanced |
| Hypertension | SMD = 0.19 | SMD = 0.07 | 0.07 | Balanced |
| Smoking | SMD = 0.21 | SMD = 0.04 | 0.04 | Balanced |
| Low fT3 OR (matched) | - | 2.73 (1.08-6.92) | P = 0.034 | Significant |

# 3. Continuous fT3 and Discrimination Analysis

The sex-stratified regression and the fT3 quartile dose-response analysis are presented in main Table 2 and as panels B and C of main Figure 3. Only the continuous fT3 model and ROC analysis are reported below.

**Supplementary Table S5.** Continuous fT3 and UIA presence (UIA vs CTRL)

*OR per 1 pmol/L decrease in fT3. Multivariable logistic regression adjusted for age, sex, hypertension, smoking, and alcohol abuse.*

| Variable | N | OR (95% CI) | P value | Test |
| --- | --- | --- | --- | --- |
| fT3 (per pmol/L decrease) | 242 | 1.42 (0.97-2.08) | 0.068 | Wald test |
| Age (per year) |  | 0.99 (0.97-1.01) | 0.471 | Wald test |
| Sex (female) |  | 5.38 (2.97-9.76) | <0.001 | Wald test |
| Hypertension |  | 1.16 (0.64-2.10) | 0.633 | Wald test |

**Supplementary Table S6.** ROC analysis: nested model comparison (UIA vs CTRL)

*AUC, area under the receiver operating characteristic curve; LR, likelihood ratio; NS, not significant. ΔAUC relative to Model 1. Adding fT3 to the base model improved discrimination by 0.038 AUC units (LR test P = 0.064); the addition of TSH and fT4 provided no further incremental value (AUC 0.728), confirming that fT3 is the primary discriminating thyroid parameter. ROC curves are shown as panel D of main Figure 3.*

| Model | AUC | ΔAUC | LR test P | Test |
| --- | --- | --- | --- | --- |
| M1: Age + Sex + HTN | 0.693 | - | - | - |
| M2: M1 + fT3 | 0.731 | 0.038 | 0.064 | LR test |
| M3: M2 + TSH + fT4 | 0.728 | 0.035 | NS | LR test |

# 4. Additional Subgroup Analyses (UIA vs CTRL)

**Supplementary Table S7.** Hypertension x fT3 interaction analysis (UIA vs CTRL)

*Wald test for fT3 x hypertension product term. The interaction was not significant (P = 0.781), indicating that the effect of low fT3 on UIA presence does not differ between hypertensive and normotensive patients.*

| Subgroup | N | fT3 OR (95% CI) | P value | Interaction P |
| --- | --- | --- | --- | --- |
| Hypertensive | 108 | 1.74 (1.01-2.98) | 0.046 |  |
| Normotensive | 134 | 1.55 (0.87-2.78) | 0.137 | 0.781 |

**Supplementary Table S8.** Levothyroxine sensitivity analysis (extended) (UIA vs CTRL)

*N = 33 levothyroxine users excluded. Continuous fT3 model (OR per 1 pmol/L decrease). Excluding these patients produced a minimally attenuated OR (1.42 to 1.36).*

| Model | N | fT3 OR (95% CI) | P value | Test |
| --- | --- | --- | --- | --- |
| Full cohort (continuous fT3) | 242 | 1.42 (0.97-2.08) | 0.068 | Wald test |
| Excl. levothyroxine users | 209 | 1.36 (0.91-2.04) | 0.134 | Wald test |

**Supplementary Table S9.** Age-stratified logistic regression (UIA vs CTRL)

*Adjusted for sex, hypertension, smoking, and alcohol abuse. Age x fT3 interaction tested via Wald test. Low fT3 was significantly associated with UIA in younger patients (<50 years; OR 2.31, P = 0.032) but not in older patients (≥50 years; OR 1.23, P = 0.358); however, the interaction was not statistically significant (P = 0.675).*

| Subgroup | N | fT3 OR (95% CI) | P value | Interaction P |
| --- | --- | --- | --- | --- |
| Age <50 years | 88 | 2.31 (1.07-4.96) | 0.032 |  |
| Age ≥50 years | 154 | 1.23 (0.79-1.93) | 0.358 | 0.675 |

**Supplementary Table S10.** Thyroid-state classification under institutional versus guideline-based TSH thresholds

*Values are patients classified as subclinical hypothyroid, n/N (%), where N is the number with an available TSH measurement. Moving from the institutional threshold of 7.78 to the guideline threshold of 4.5 newly classified 9 RIA, 2 UIA and 1 control patient.*

| TSH upper limit (mU/L) | RIA | UIA | CTRL |
| --- | --- | --- | --- |
| 7.78 (institutional) | 5/599 (0.8) | 0/205 (0.0) | 1/255 (0.4) |
| 4.5 (guideline) | 14/599 (2.3) | 2/205 (1.0) | 2/255 (0.8) |

**Supplementary Table S11.** Free triiodothyronine by control diagnostic category

*Low fT3 is defined as below 3.5 pmol/L. The low-fT3 burden within the control group is concentrated in the suspected-stroke and no-defined-pathology categories, both resting on small numbers of measured patients, whereas Parkinson disease controls show a low-fT3 prevalence of 3.6 per cent. Because the low-fT3 counts in the suspected-stroke and no-defined-pathology categories rest on only 9 and 19 patients with a measured fT3, these subgroup estimates are imprecise and are presented as descriptive only.*

| Diagnostic category | n | fT3, mean ± SD (n measured) | Low fT3, n/N (%) |
| --- | --- | --- | --- |
| Idiopathic Parkinson disease | 111 | 4.72 ± 0.74 (56) | 2/56 (3.6) |
| Suspected stroke | 57 | 3.54 ± 1.16 (9) | 5/9 (55.6) |
| Epilepsy | 24 | 4.28 ± 0.75 (14) | 1/14 (7.1) |
| Cervical dystonia | 11 | 4.53 ± 0.80 (9) | 1/9 (11.1) |
| No defined pathology | 55 | 4.17 ± 1.20 (19) | 6/19 (31.6) |

**Supplementary Table S12.** Regression diagnostics for the UIA versus control model

*Linearity of the logit was assessed with the Box-Tidwell approach and influence with Cook distance and standardised residuals. No assumption violation was detected. The small number of low-fT3 exposed UIA cases is the factor limiting the stability of the low-fT3 estimate, and these analyses are hypothesis-generating.*

| Diagnostic | Result | Interpretation |
| --- | --- | --- |
| Box-Tidwell linearity of the logit, age | Interaction P = .940 | Assumption satisfied |
| Box-Tidwell linearity of the logit, fT3 | Interaction P = .436 | Assumption satisfied |
| Cook distance, maximum | 0.243 | No extreme influential point |
| Observations exceeding 4/n (0.017) | 16 of 242 | Reviewed; conclusions unchanged |
| Complete cases in the model | 242 | Six covariates fitted |
| Low-fT3 exposed UIA cases | 6 | Limiting quantity for stability |

**Supplementary Table S13.** Availability of thyroid measurements by group

*Values are n (%) of the group total. fT3 availability differed markedly between groups (73.7 per cent in UIA versus 40.5 per cent in RIA and 41.5 per cent in controls; chi-square P < .001). Because cohort entry required an available thyroid panel and the decision to measure fT3 was made clinically, this is consistent with indication bias.*

| Group | Total | TSH available | fT3 available | fT4 available |
| --- | --- | --- | --- | --- |
| RIA | 629 | 599 (95.2) | 255 (40.5) | 277 (44.0) |
| UIA | 213 | 205 (96.2) | 157 (73.7) | 174 (81.7) |
| CTRL | 258 | 255 (98.8) | 107 (41.5) | 116 (45.0) |
